# Towards machine learning-based quantitative hyperspectral image guidance for brain tumor resection

**DOI:** 10.1038/s43856-024-00562-3

**Published:** 2024-07-04

**Authors:** David Black, Declan Byrne, Anna Walke, Sidong Liu, Antonio Di Ieva, Sadahiro Kaneko, Walter Stummer, Tim Salcudean, Eric Suero Molina

**Affiliations:** 1 Department of Electrical and Computer Engineering, University of British Columbia, Vancouver, BC Canada; 2 Department of Neurosurgery, University Hospital Münster, Münster, Germany; 3 Computational NeuroSurgery (CNS) Lab, Macquarie Medical School, Faculty of Medicine, Health and Human Sciences, Macquarie University, Sydney, NSW Australia; 4 Department of Neurosurgery, Hokkaido Medical Center, National Hospital Organization, Sapporo, Japan

**Keywords:** Diagnostic markers, Computational neuroscience, CNS cancer

## Abstract

**Background:**

Complete resection of malignant gliomas is hampered by the difficulty in distinguishing tumor cells at the infiltration zone. Fluorescence guidance with 5-ALA assists in reaching this goal. Using hyperspectral imaging, previous work characterized five fluorophores’ emission spectra in most human brain tumors.

**Methods:**

In this paper, the effectiveness of these five spectra was explored for different tumor and tissue classification tasks in 184 patients (891 hyperspectral measurements) harboring low- (*n* = 30) and high-grade gliomas (*n* = 115), non-glial primary brain tumors (*n* = 19), radiation necrosis (*n* = 2), miscellaneous (*n* = 10) and metastases (*n* = 8). Four machine-learning models were trained to classify tumor type, grade, glioma margins, and IDH mutation.

**Results:**

Using random forests and multilayer perceptrons, the classifiers achieve average test accuracies of 84–87%, 96.1%, 86%, and 91% respectively. All five fluorophore abundances vary between tumor margin types and tumor grades (*p* < 0.01). For tissue type, at least four of the five fluorophore abundances are significantly different (*p* < 0.01) between all classes.

**Conclusions:**

These results demonstrate the fluorophores’ differing abundances in different tissue classes and the value of the five fluorophores as potential optical biomarkers, opening new opportunities for intraoperative classification systems in fluorescence-guided neurosurgery.

## Plain language summary

Complete surgical removal of some primary brain tumors is difficult because it can be hard to distinguish the edge of the tumor. We evaluated whether the edges of tumors and the tumor type and grade can be more accurately determined if the tumor is imaged using many different wavelengths of light. We used measurements taken from the tumors of people undergoing brain tumor surgery and developed machine-learning algorithms that could predict where the edge of the tumor was. The methods could also provide information about the type and grade of the brain tumor. These classifications could potentially be used during operations to remove brain tumors more accurately and thus improve the outcome of surgery for people with brain tumors.

## Abstract (web summary)

Black et al apply machine learning techniques to a large dataset of endmember abundance vectors derived from hyperspectral fluorescence measurements of brain tumors. They show that it is possible in this way to classify various tissue types, grades, and biomarkers with a high degree of accuracy.

## Introduction

Surgical resection of malignant glioma is complex, and recurrences are the rule rather than the exception, leading to patients’ poor prognosis^1^. This is partly due to poorly differentiated tumor tissue, especially at infiltrating margins, closely resembling healthy tissue during surgery^2^. To address this problem, 5-aminolevulinic acid (5-ALA)-induced protoporphyrin IX (PpIX) fluorescence guidance has been established as a surgical adjunct in neurosurgery. The complete resection rate for contrast-enhancing tumors increased, in the initial approval study from 2006, from 36% operated under white light only to 65% for resections with fluorescence guidance^2^. Improvements in neuroimaging and different surgical adjuncts, such as intraoperative ultrasound, pre- and intra-operative brain mapping, and monitoring techniques, amongst others, currently allow for over 95% complete resection rates, whenever feasible^3^. Consequently, PpIX fluorescence is widely used in the resection of high-grade gliomas. Furthermore, it is the subject of research in low-grade gliomas^4–7^, meningiomas^8–11^, and other brain tumors^12^, as well as in oral cancer^13,14^, bladder cancer^15,16^, and skin cancer^17^. Additionally, 5-ALA is used for photodynamic therapy^18^, to treat skin and other brain malignancies^19^.

Fluorescence is achieved by administering 5-ALA orally prior to surgery^1^, which is metabolized to PpIX, a precursor in heme biosynthesis. The mechanisms leading to the selective accumulation of PpIX in glioma cells^20^ are not entirely understood^1,11,21^. Several explanations have been proposed, including disruption of the blood-brain barrier, as commonly observed in high-grade gliomas (HGG), which is otherwise non-permeable to 5-ALA^1,7,22^. Reduced activity of ferrochelatase, which would otherwise metabolize PpIX^15,21,23^, and changes in the tumor microenvironment affecting 5-ALA uptake and PpIX efflux^24–26^ may play a part as well. However, PpIX is present in increased concentrations 7–8 hours after 5-ALA administration in HGG^27^. PpIX fluoresces after illumination with intense 405 nm (blue) light. Absorption of such a photon lifts the molecule to an excited state, decreasing slightly through vibrational relaxation before returning to its ground state and emitting a second photon^28^. Due to the decrease in energy before the second transition, the emitted photon has a longer wavelength of around 634 nm (red). This energy change called the Stokes shift, allows the red fluorescing areas to be differentiated from the otherwise blue-reflecting tissue^29^.

With optical highpass filters such as the BLUE400 and BLUE400 AR systems (Carl Zeiss Meditec AG, Oberkochen, Germany) that block the relatively intense reflected blue light but transmit the red fluorescence, surgeons can differentiate 5-ALA positive tumor from surrounding tissue^30,31^. This has led to widespread adoption. However, the proven increased resection rates are only for malignant glioma. Unfortunately, many lower-grade gliomas and even some with high-grade regions exhibit low PpIX accumulation and do not visibly fluoresce. Furthermore, autofluorescence makes it impossible to discern the diminished PpIX fluorescence even when imaging sensitivity is increased because it shares the same spectral range as the PpIX emission^32–35^.

Hence, quantitative spectroscopic systems have been developed that measure the emission spectra and separate PpIX from autofluorescence through a priori knowledge of the fluorophores present and their individual emission spectra^35–37^. These devices have been used primarily in research and consist of either point probes^5,8,37–39^ or wide-field hyperspectral^12,40,41^ devices. In addition to potential future intraoperative use to help distinguish tumors, spectroscopy can answer many questions about different diseases^23,36^, how best to treat them^27,40,41^, and how to improve imaging systems for future intra-operative integration^12,42^.

One potential use of such a system is to distinguish between different tissue types. Several classifications are commonly applied to brain tumors, including the tumor type (e.g., glioma, metastasis, medulloblastoma, and others^43^). Additionally, the differentiation of tumor from non-tumor or necrotic tissue (e.g., radiation necrosis, which can radiologically mimic tumor progression) is relevant in neuro-oncology and neurosurgery. Different tissue types differ substantially in behavior and prognosis, so their correct identification is paramount. Several studies have classified brain tumor type in MR images^44,45^, but the same has not been attempted with fluorescence.

Furthermore, gliomas are classified according to their histological, genetic features, and biological behavior^46^. They are categorized as grade I-IV by the World Health Organization (WHO) classification system, where traditionally, I and II are considered low grade, and III and IV are high grade^47^, indicating a bad prognosis^48^. Molecular parameters, i.e., isocitrate dehydrogenase (IDH)-mutation, O6-methylguanine-DNA-methyltransferase (MGMT), among others, assist in further subclassification of these tumors^49^. We aim to predict tumor molecular characteristics, such as IDH mutations or genetic aberrations related to malignancy, by correlation with the spectral signature of tumors. Such knowledge would directly result in changes to surgical strategy. IDH mutations cause a shift in enzymatic activity, converting α-ketoglutarate to 2-hydroxyglutarate and inhibiting α-KG-dependent enzymes. This leads to metabolic reprogramming, hinders cell differentiation, and initiates tumorigenesis^50^. Small molecule inhibitors can reverse this process, making knowledge of IDH mutation status crucial for guiding their targeted use in treatment. Knowing a tumor’s grade or molecular characteristics could also help make decisions during and after surgery. The tumor types are shown in Supplementary Fig. 2. Some studies have performed automatic classification of tumor grade using convolutional neural networks (CNN) on magnetic resonance images^51^ and digitized histopathology slides^52^, but not through fluorescence.

Additionally, one of the primary difficulties of resection of grade II, III, and IV gliomas is the infiltrative nature of the tumors. Around the solid tumor portion, there is a region of infiltrative margin characterized by decreased tumor cellularity transitioning to healthy tissue^53^. To delineate the margins, guidance from pre-operative MRI images is commonly used in intraoperative neuronavigation systems^54^, but its accuracy suffers from factors such as brain shift^55^. Differentiating more accurately between margin regions would significantly enhance the effectiveness of surgical resection of glioma and patients’ outcomes^56,57^. Leclerc et al. presented the first work applying machine learning (ML) to neurosurgical fluorescence spectroscopy, classifying different tissues based on principal component analysis (PCA) of the fluorescence spectra^58^, achieving 77% accuracy on 50 samples.

Although previous work has correlated PpIX fluorescence with WHO grade and tumor margins^59^, it is not well understood how different tumor types affect the abundances of the different fluorophores^60^. Through recent characterization of their basis spectra^35^, we can now precisely analyze the abundances of the major fluorophores in hyperspectral fluorescence images. Hence, it is possible to study how the two photo-states of PpIX and the autofluorescence from flavins (i.e., flavin adenine dinucleotide), NADH, and lipofuscin are affected by the tissue type. This paper performs four classification tasks using fluorescence spectroscopy, aiming to use the abundances of the five fluorophores to differentiate between (1) tumor and tissue types (e.g., glioblastoma, meningioma, etc.), (2) WHO grades, (3) between solid tumor, infiltrating zone, and reactively altered non-tumor brain tissue, and (4) between IDH-mutant and IDH-wildtype glioma. Using random forests and multilayer perceptron, the classifiers achieve average test accuracies of 84–87%, 96.1%, 86%, and 91%, respectively, thus demonstrating the fluorophores’ differing abundances in different tissue classes and the value of the five fluorophores as potential optical biomarkers. This invites further research into intraoperative classification systems in fluorescence-guided neurosurgery.

## Methods

### Device and Dataset

A hyperspectral imaging device was used, as previously described^27,41,59^, and outlined below. Patients received 5-ALA orally at a dose of 20 mg/k.g. b.w., four hours prior to anesthesia induction for tumor resection surgery, as per routine practice in cases of suspected malignant gliomas. All Patients consented to the use of 5-ALA for fluorescence-guided resection. The tumor entity is not known prior to surgery. Thus, occasionally, patients harboring distinct tumor types also received 5-ALA. All ex vivo data collection was carried out with informed consent, complied with institutional guidelines, and was approved by the ethical committee of the University of Münster.

**Fig. 1:**
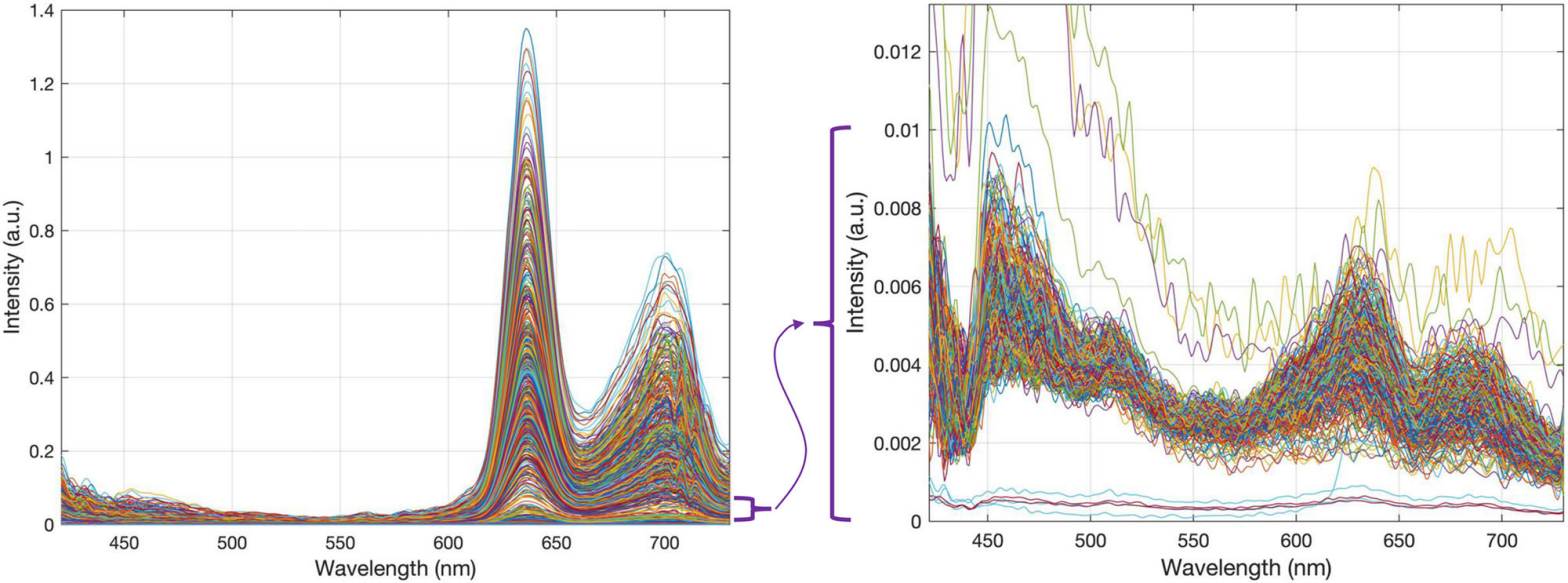
Typical Example Measured Spectra. The plot of representative 1000 spectra sampled randomly from the complete dataset (left). Furthermore, we added a zoomed-in view of some typical weak spectra, where the different autofluorescence contributions can be visualized, not just PpIX.

Tissue samples were resected during surgery and measured directly ex vivo on a petri dish. The sample was first illuminated with blue light (405 nm LED), then with no light for recording background noise, and finally with broadband white light. During each illumination phase, the emitted light from fluorescence and the reflected light were gathered in the objective lens (OPMI Pico - Carl Zeiss AG, Oberkochen, Germany) and routed to a scientific metal oxide semiconductor (sCMOS - PCO.Edge, Excellitas Technologies GmbH, Wiesbaden, Germany) camera through a series of optical filters. The first filters removed the intense blue reflected light. Next, a liquid crystal tunable bandpass filter (LCTF - Meadowlark Optics, Frederick, CO) transmitted a narrow spectral band of the emitted light to the sCMOS. The LCTF was swept across the visible range (420–730 nm) in 3–5 nm increments, during each of which a grayscale image was captured by the sCMOS. In this way, a hyperspectral data cube containing all the spectral and spatial information was generated. Any pixel can be selected, and an emission spectrum extracted for that pixel from the cube. Figure 1 shows 1000 examples of typical measured spectra. During blue-light illumination, the fluorescence spectra were captured. The white-light illuminated data cube was used for dual-band normalization^42^, and the no-light spectra were used to remove the dark noise of the camera sensor from the images. Total acquisition time with 500 ms exposure time for each image is 3–4 minutes. This ensures a good signal-to-noise ratio, even with very little fluorescence. Each image was 2048 × 2048 pixels with 47.62 pixels per mm.

This device was used for ex vivo analysis of tumor biopsies at the University Hospital Münster, Germany. Typical samples had an average diameter of 288 pixels, or 6 mm, and ranged from approximately 4 to 10 mm. The resulting data set was used in this study to explore the effect of different classifications of tumors on the presence of the five main fluorophores. In particular, we analyzed tumor type, WHO grade, and whether the sample is from solid tumor, infiltrating zone, or non-tumor. The classes and number of samples are shown below and were chosen based on the availability of patient data. Many samples were measured before the updated WHO classification (CNS5, 2021)^47^ and thus followed the previous 2016 WHO classification system^49^.

*Tissue Type Classification:* (n = 632 biopsies)

Pilocytic astrocytoma (PA; *n* = 5), diffuse astrocytoma (DA; *n* = 57), anaplastic astrocytoma (AA; *n* = 51), glioblastoma (GB; *n* = 410), grade II oligodendroglioma (OD; *n* = 24), ganglioglioma (GG, *n* = 4), medulloblastoma (MB; *n* = 6), anaplastic ependymoma (AE; *n* = 8), anaplastic oligodendroglioma (AO; *n* = 4), meningioma (MN; *n* = 37), metastasis (MT; *n* = 6), radiation necrosis (RN; *n* = 20).

*Margin Classification:* (*n* = 288 biopsies)

Solid tumor (ST; *n* = 131), infiltrating zone (IZ; *n* = 57), reactively altered brain tissue (RABT; *n* = 100).

*WHO Grade Classification:* (*n* = 571 biopsies)

Grades I (*n* = 9), II (n = 84), III (*n* = 57), IV (*n* = 421).

*IDH Classification:* (*n* = 411 biopsies)

IDH-mutant (*n* = 126), IDH-wildtype (n = 285)

Note that the RABT class is the tissue outside the infiltration zone that cannot be classified as a solid tumor or infiltration zone. Intraoperatively, it is imperative to differentiate this from tumor tissue, which can be viewed as healthy tissue. It is otherwise unethical to biopsy healthy brain tissue.

**Fig. 2:**
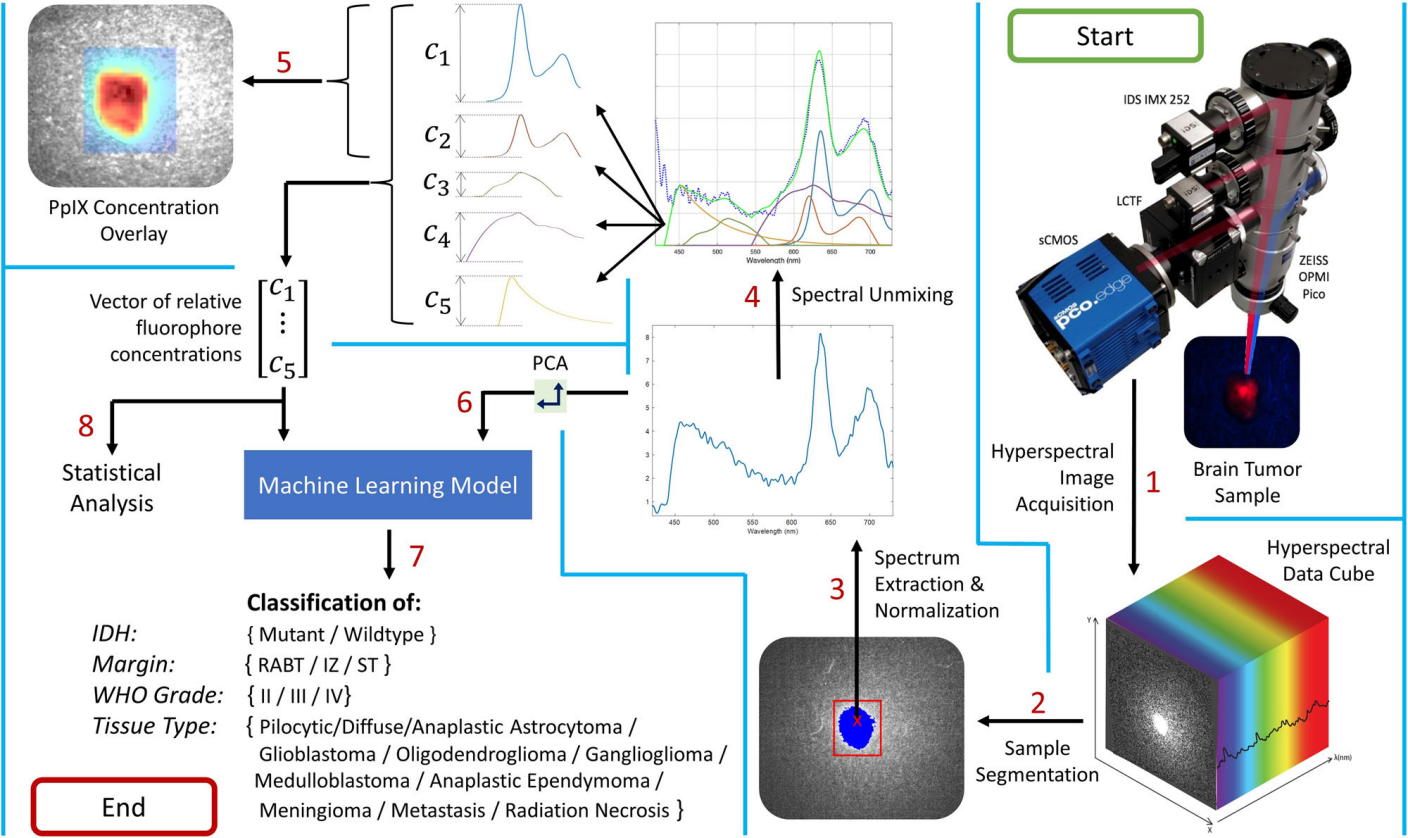
Overview of the device and method. This shows the steps of the data processing, from top right to bottom left. The imaging device first captures a hyperspectral data cube from which the ex vivo tumor tissue is segmented and the corresponding spectra are extracted, processed, and unmixed into abundance vectors or dimensionally reduced using PCA. The abundances can be used, for example, to create a PpIX overlay plot or perform statistical analyses. In this study, they are used as inputs to machine learning models to classify tumor type, margins, WHO grades, and IDH mutation.

The biopsies varied in size and shape, but approximately 100–1000 spectra were extracted from each. Regions of 10 × 10 pixels were averaged to produce one spectrum for noise reduction, and the regions were non-overlapping to ensure each data point was independent. For a given classification task, spectra were sampled randomly from the tumor portions of the biopsies. To avoid inadvertently sampling from the background glass slide, the tumor was first segmented automatically in MATLAB using the 634 nm fluorescence image. The result is shown in step 2 of Fig. 2; the resulting masks were also checked manually.

The raw fluorescence spectra were corrected, normalized, and unmixed as described previously^12,35^. Each fluorescence spectrum is assumed to consist of a linear combination of five fluorophores whose spectra are known a priori. These basis spectra are the two photo-states of PpIX (denoted PpIX634 and PpIX620)^36,37^, NADH, lipofuscin, and flavins^35^. The example measured and the basis spectra are described and shown in Fig. 2 and in previous work^35^. The unmixing calculates the abundances of the five spectra by minimizing the squared error between the measured spectrum and a linear combination of the five spectra. If the basis spectra are combined as columns in a 310 × 5 matrix $B$, the measured spectrum is the 310 × 1 vector ${{{{{\boldsymbol{y}}}}}}$, and the 5 × 1 vector of abundances is ${{{{{\boldsymbol{c}}}}}}$, then we find ${{{{{\boldsymbol{c}}}}}}$ as

$$c={{{{{{\rm{argmin}}}}}}}_{c}\left\{\frac{1}{2}||y-B{c}||^{2}\right\}$$

using non-negative least squares. This is illustrated in steps 3–5 of Fig. 2.

### Machine learning approach

With the processed data, we tried several ML classification models using Python and experimented with different hyperparameters. The various types are listed in Table 1.

**Table 1:** Explored Classifier Algorithms and Hyperparameters

Pixels per Sample	[1, 2, 3]		
Samples per Class	Tumour Type	Margin	WHO Grade
	[300, 500, 800]	[1000, 3000, 5000]	[500, 1500, 3000]
Random Forests^69^	Number of trees: [50, 75, 100, 125, 150] Splitting Criteria: [Gini, Entropy, Log loss] Minimum samples to split: [2, 3, 4] Maximum features per tree: [Square root (sqrt), log2, None]		
KNNs^70^	Number of neighbors: [3, 5, 7, 9] Weights: [Uniform, Distance] p: [1, 2]		
SVM^71^	Kernel: [Radial Basis Function (RBF), Linear, Polynomial, Sigmoid]		
MLP^72^	Number of hidden layers: [1,2,3] Number of neurons: [25, 50, 100, 150] Activation: ReLU (Rectified linear unit) Solver: [Adam, Limited-memory BFGS (LBFGS)] Nesterov Momentum: 0.9		
AdaBoost^73^	Number of estimators: 50 Learning rate: 1.0 Algorithm: SAMME.R		

Multilayer perceptron (MLP), support vector machine (SVM), and k-Nearest Neighbor (KNN) classifiers are described in the papers cited in the table.

For a given biopsy, instead of using the abundances inferred from only one pixel, it could be more informative to use those of two or more nearby pixels. This is attempted in the *pixels per sample* hyperparameter. Further, to avoid bias, the same number of samples of each class were used to train the classifiers. Since some classes had fewer samples, we varied the number of samples per class. A class with fewer samples than the samples per class hyperparameter was excluded from the classification. Lower numbers of samples per class allowed more classes to be included in the classification. However, more significant numbers of samples per class allowed for more effective training of the classifiers, though on fewer classes. Classes with few biopsies, including anaplastic oligodendroglioma and ganglioglioma, had to be excluded from some tests.

The dataset was split 80/20 into a training and testing set. Hyperparameter and model tuning were carried out using 5-fold cross-validation on the training set to mitigate optimization bias. In total, 195 models were trained and compared for each classification task. For each of the four classification tasks, nine datasets were used with varying pixels per sample and samples per class. Finally, the best model was selected for each category and evaluated with the test set. The receiver operating characteristic curves (ROC) were determined to evaluate the multiclass classifiers, and the area under the curve (AUC) was computed. Accuracy was calculated as the number of correctly classified samples divided by the total number of samples. Confusion matrices were used to determine which classes performed better than others.

We also explored whether the calculated abundances were the most informative space to characterize the biopsies. Instead of relying on the five basis spectra, we repeated the above experiments with the mathematically optimal projection of the spectra onto a 5-dimensional hyperplane using principal component analysis (PCA). We used the same number of pixels per sample, samples per class, and all other hyperparameters as above. We also visualized the different classes using only the first two or three principal components in a scatter plot. PCA finds a set of orthogonal axes, which maximizes the variance. Depending on assumptions about the signal and noise distributions, this maximizes the mutual information between the real signal and the dimensionally-reduced output^61^. Nonetheless, some information was lost. To quantify how well PCA represents the original data, we utilized the variance explained parameter. Given a data matrix of fluorescence spectra, a covariance matrix whose diagonal elements (the variances) sum to the overall variability can be computed. When performing n-component PCA, only the n principal components corresponding with the n largest eigenvalues were kept. All the eigenvalues sum to the overall variability, so by choosing only the n-largest, we lost some of the information contained in the data. In particular, the ratio of the sum of the n selected eigenvalues over the total variability was called the variance explained. It effectively yielded the percent of total information the chosen principal components represented.

#### Statistics and reproducibility

Statistical analyses were performed to determine correlations and statistical significance. This analysis was performed in MATLAB using the two-sample Kolmogorov-Smirnov test. Python and SciKit-Learn were used for the machine learning algorithms. The entire process, from imaging to data processing and extraction to analysis, is shown in Fig. 2, and the raw results and all p values are found in Supplementary Data 1 and 2, respectively.

### Reporting summary

Further information on research design is available in the Nature Portfolio Reporting Summary linked to this article.

## Results

As explained in detail in the Methods section, four classification tasks were explored. Tumor types belonged to the following classes: Pilocytic astrocytoma (PA), diffuse astrocytoma (DA), anaplastic astrocytoma (AA), glioblastoma (GB), grade II oligodendroglioma (OD), ganglioglioma (GG), medulloblastoma (MB), anaplastic ependymoma (AE), anaplastic oligodendroglioma (AO), meningioma (MN), metastasis (MT), and radiation necrosis (RN). Tumor margin classification was performed on three classes: solid tumor (ST), infiltrating zone (IZ), and reactively altered brain tissue (RABT). Reactively altered brain tissue can be considered as healthy tissue. IDH status (mutated and wildtype) was further assessed. Finally, WHO grades II, III, and IV were considered. After hyper-parameter tuning using cross-validation on the training dataset, the following results and metrics were calculated using the test set.

### Visualization

**Fig. 3:**
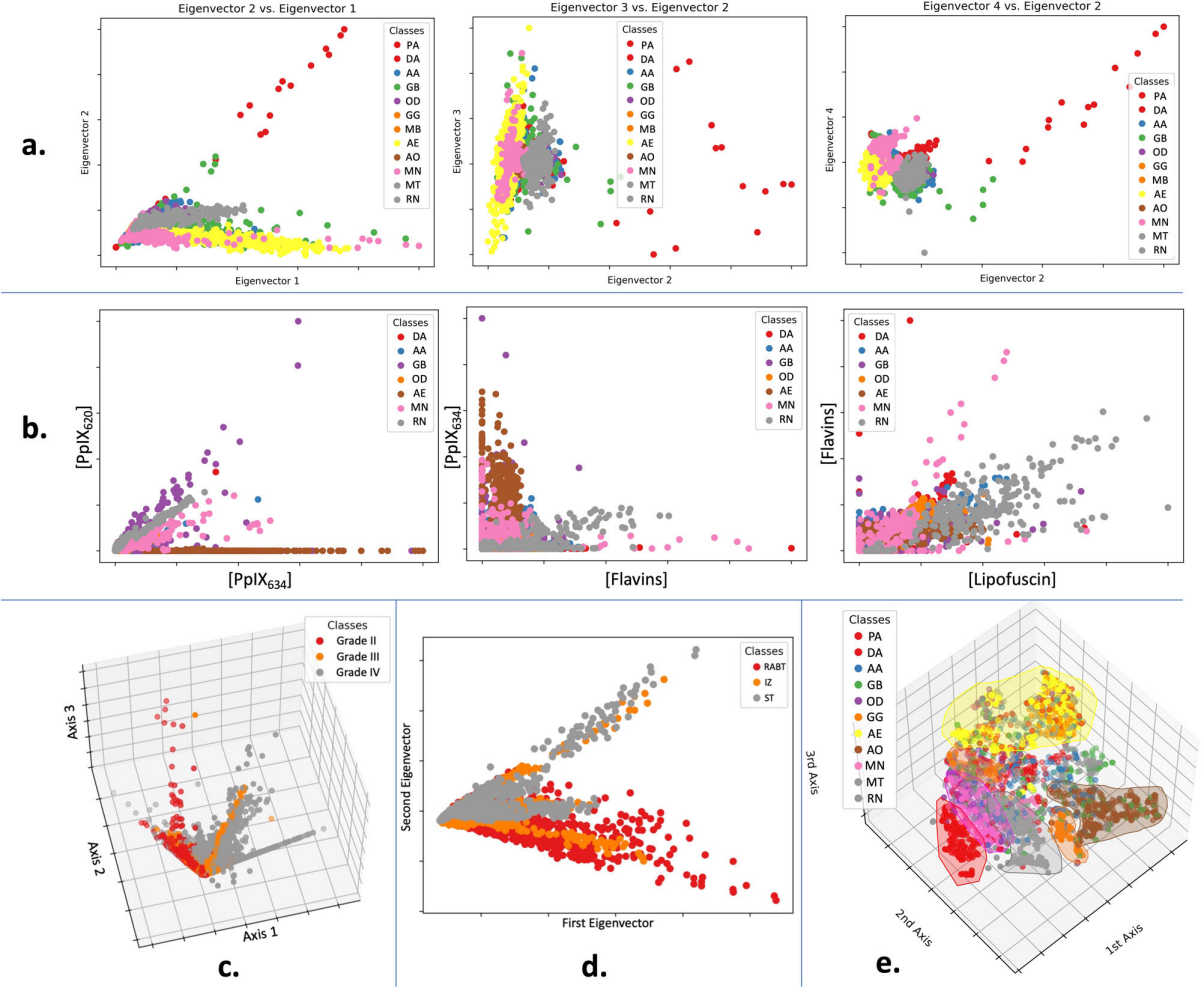
Visualizations of tumor data for different classifications. Several classes are visually distinguishable or nearly so despite being very low-dimensional representations, which is promising for ML classification. Panels **a** and **b** compare the effectiveness of PCA versus fluorophore abundances for visualizing the classes, with similar results. This suggests that the fluorophore abundances are already close to the most informative features for representing the data. Subplots **c** and **d** show PCA on WHO grade and tumor margin respectively, while the others show tumor type. Finally, panel **e** shows a 3-component T-SNE on tumor type. Here, we see distinctions between some groups, though others, including glioblastoma and astrocytoma, remain very mixed. All axes are in arbitrary units. WHO grade I and some tumor types are excluded due to relatively small sample size and unbalanced datasets. The classification abbreviations are as follows: Pilocytic astrocytoma (PA), diffuse astrocytoma (DA), anaplastic astrocytoma (AA), glioblastoma (GB), grade II oligodendroglioma (OD), ganglioglioma (GG), medulloblastoma (MB), anaplastic ependymoma (AE), anaplastic oligodendroglioma (AO), meningioma (MN), metastasis (MT), radiation necrosis (RN), solid tumor (ST), infiltrating zone (IZ), reactively altered brain tissue (RABT). Several of these tumors were operated on before 2021 and are therefore classified according to the older WHO classification system, which is no longer in use.

Since the five known fluorophore abundances create a 5-dimensional space, it is impossible to visualize the data and distinguish patterns manually. Studying scatter plots of two fluorophores at a time would be feasible, but this would be a reductionistic approach drawing a very limited picture. Instead, we used PCA for dimensionality reduction, which results in some information loss. The exact amount of information lost for each classification is shown in Table 2. However, PCA fails if the data forms a non-linear manifold. One alternative that captures this more effectively is t-distributed stochastic neighbor embedding (t-SNE). This was also used to better visualize the data. Some of the more informative plots are shown in Fig. 3.

**Table 2:** Variance Explained from 2 to 5-Dimensional PCA

PCA Dimension	Tumor Type	Margin	WHO Grade
2	79%	79%	85%
3	87%	89%	93%
4	94%	95%	95%
5	97%	99%	96%

A value of 100% means that all variance is accounted for. With five values, almost no information is lost, indicating that the 5 primary fluorophores most likely cause the measured emissions.

Though drawing conclusions from the visualizations in Fig. 3 is difficult, it shows that some classes can be visually distinguished from others with reasonable confidence, even in dimensionally reduced form. Furthermore, Fig. 3a and b show that the fluorophores and PCA give similar information, suggesting the fluorophore abundances’ utility as informative input features to ML classifiers. Table 2 additionally demonstrates that almost all the spectral information is stored in five dimensions, which lends further credence to the five a priori basis spectra^35^.

### Tissue type classification

Since the tissue type data is categorical, it is not possible to determine linear correlations. However, pairwise Kolmogorov-Smirnov tests were used to test the null hypothesis for each fluorophore: abundances from each pair of classes come from the same distribution. All available spectra were used in the significance tests for each class, which ranged from 200 to over 5000, depending upon the class. The chosen test (kstest2.m in MATLAB) does not require equal sample sizes. All class pairs were significantly different (*p* < 0.01) across all the fluorophores except those listed in Supplementary Table 1. Thus, even if a pair of classes did not differ significantly in one fluorophore, it did in the remaining four. All p values are found in Supplementary Data 2.

Both multilayer perceptrons (MLPs) and random forests performed well for classifying the tumor type. None of the other classifiers were effective. Performance was better with more pixels per class, partly because the models were trained on more data and partly because they had to distinguish between fewer classes. Therefore, we present the best results overall and the best results from training with all the classes.

**Fig. 4:**
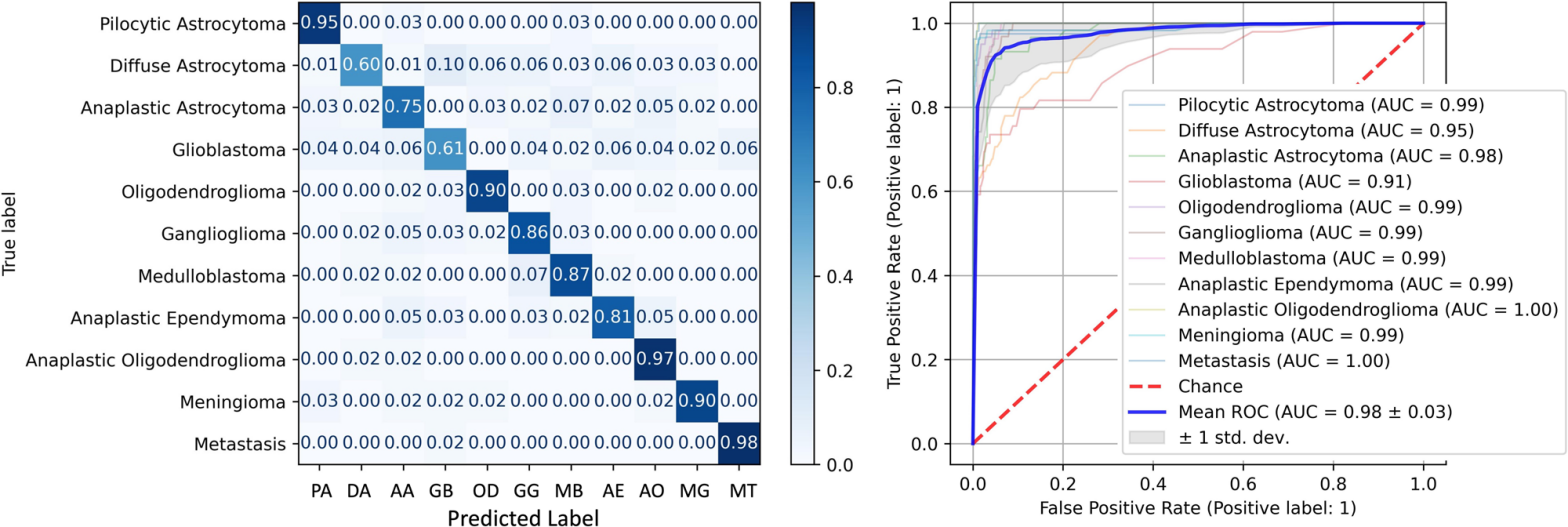
Tumor type classifier performance. Confusion matrix and ROC for the best-performing random forest classifier.

The best-performing classifier, including most classes, was a random forest model (150 trees, 500 samples per class, 3 pixels per sample, square root of the number of features sqrt(n_features) features per tree, cross-entropy splitting criterion). The average accuracy was 83.6%, with an AUC of 0.98 in classifying between all classes. Figure 4 shows this classifier’s confusion matrix and multiclass ROC.

This shows outstanding performance for some classes and relatively poor for others. In particular, GB, DA, and AA have low classification accuracy, and GB is frequently mistaken for AE. However, AE is rare and generally easy to distinguish visually from healthy tissue, so fluorescence is not commonly used. Hence, despite its good classification accuracy, AE was removed. Additionally, this data used the 2016 WHO classification because it was partly collected before 2021. After the 2021 WHO classification, however, IDH-wildtype anaplastic astrocytomas are considered GB. Thus, these samples (*n* = 33) were re-labeled as GB, and the models were re-trained.

**Fig. 5:**
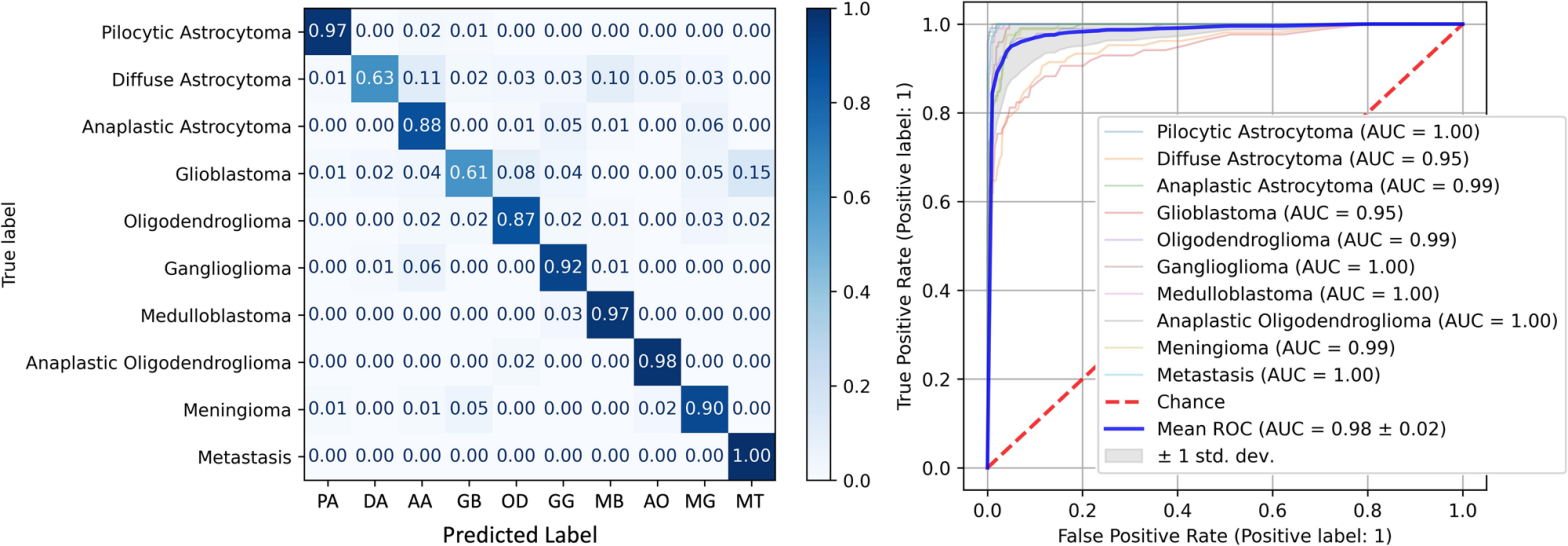
Tumor type classifier performance (No AE). Confusion matrix and ROC are used for the best-performing random forest classifier of tumor types with AE excluded, and IDH-wildtype AA relabeled as GB.

With these changes, the best-performing model used the same algorithm and hyperparameters as above but gave 87.3% accuracy and an average AUC of 0.98, as shown in Fig. 5.

We also considered classifying between higher-level groupings of tumor types (i.e., higher in the hierarchy of Supplementary Fig. 2). The classes were glioma, meningioma, medulloblastoma, and radiation necrosis; ependymoma and metastasis were excluded for small sample size. The best-performing model for this task was an MLP (150 neurons per layer, 3 hidden layers, 500 samples per class, 2 pixels per sample, Adam solver). The model achieved 89.8% accuracy and an AUC of 0.97, as shown in Supplementary Fig. 3. The glioma class is very broad, likely leading to slightly lower accuracy. Generally, however, the performance is good. The accuracy is boosted to 90.67% by using 800 samples per class, which necessitates leaving out radiation necrosis.

Therefore, tumor type does indeed have a strong effect on the five fluorophores: strong enough that it is possible to distinguish between eleven types of tumors and other tissue types with a relatively high degree of accuracy. Some gliomas, specifically GB, DA, and AA, were difficult to discern. Meningioma was also sometimes misclassified as medulloblastoma. On the other hand, many tissue types, including ganglioglioma, radiation necrosis, and, to a slightly lesser degree, medulloblastoma, could be classified with almost perfect accuracy.

Using PCA instead of fluorophore abundances to perform this analysis did not improve results. This is likely due to the fact that PCA does not seem to provide more information than the fluorophores.

### Margin classification in gliomas

The margin classification would be practically useful in an intraoperative setting. It could delineate solid tumor from infiltrating cells and non-tumor tissue. However, the distinction is relatively subjective (see Discussion section), and as shown in Fig. 3e, there may be substantial overlap between classes. Again, however, all fluorophore abundances significantly differ between every category (*p* < 0.01).

**Fig. 6:**
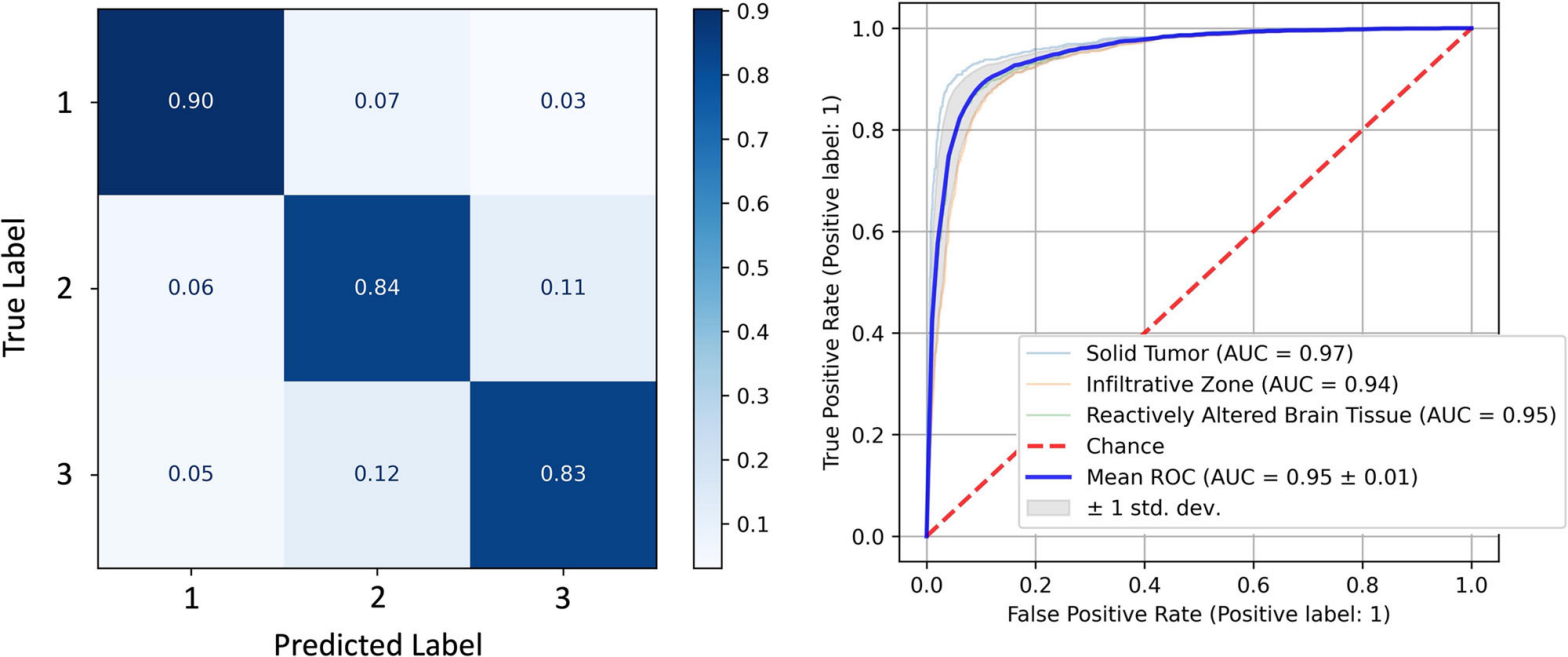
Tumor Margin Classifier Performance. Confusion matrix and ROC for the best performing MLP classifier of tumor margins. The classes are [1: Solid tumor, 2: Infiltrating zone, 3: Reactively altered brain tissue].

Consequently, a multilayer perceptron (5000 samples per class, 3 pixels per sample, 100 neurons per hidden layer, 5 hidden layers, Adam solver) distinguishes with 85.7% accuracy and a mean AUC of 0.95, as shown in Fig. 6.

It is reasonable that the infiltrating zone is the most commonly mistaken for solid tumor and reactively altered brain tumor, whereas the latter two are infrequently confused.

### WHO grade classification

**Fig. 7:**
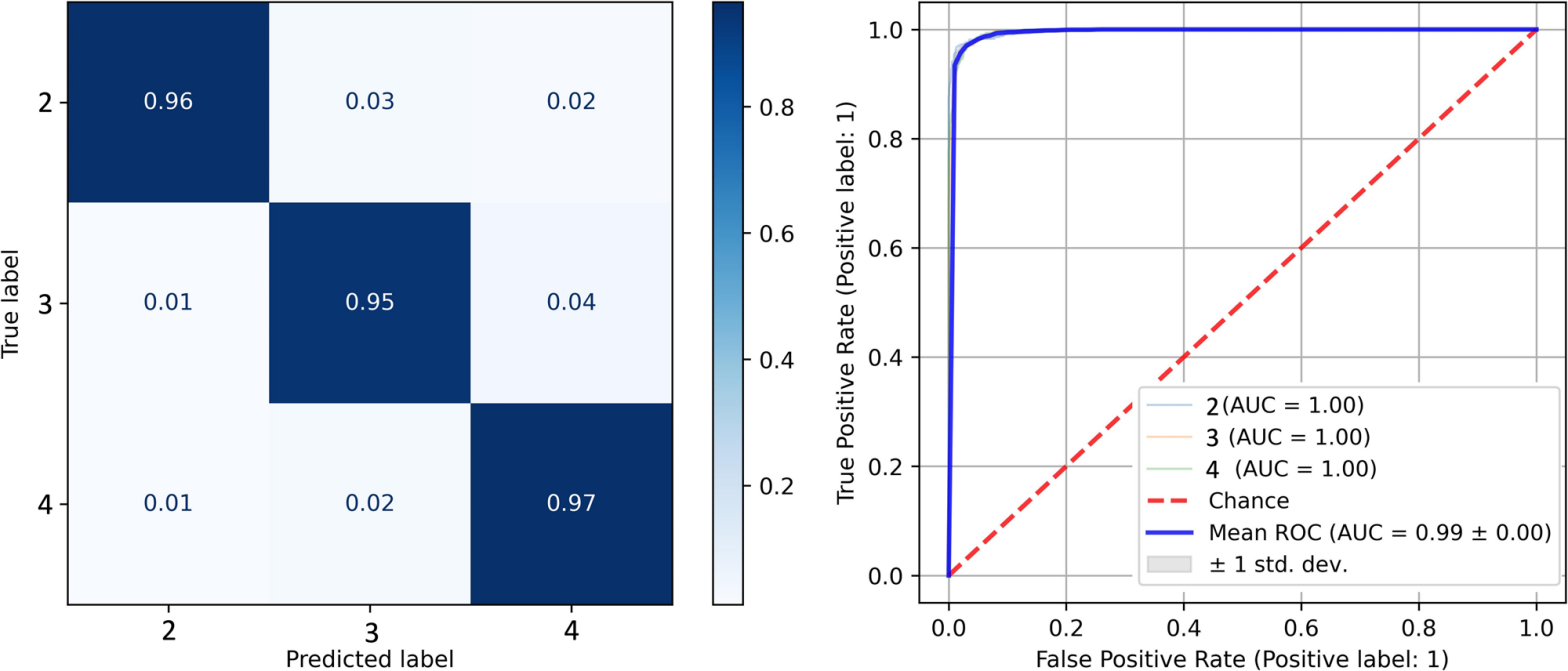
WHO Grade Classifier Performance. Confusion matrix and ROC for the best performing random forest classifier of WHO grades. The classes are [2: Grade II, 3: Grade III, 4: Grade IV].

Furthermore, knowing what WHO-grade certain tumors or tissue regions have during an operation would be valuable and further reinforce the malignant character of the tissue. In practice, many low-grade tumors have an anaplastic focus, which fluoresces, while the surrounding tumor does not. All differences in fluorophore abundances between WHO grades were found to be statistically significant (*p* < 0.01). For this task, a multilayer perceptron classifier performed best (3000 samples per class, 3 pixels per sample, 150 neurons per layer, 5 hidden layers, Adam solver), with 96.1% accuracy and a testing AUC of 0.99, as shown in Fig. 7. While WHO grades II and III were classified with good accuracy, grade III tumors were more frequently confused for grade IV.

### IDH mutation

**Fig. 8:**
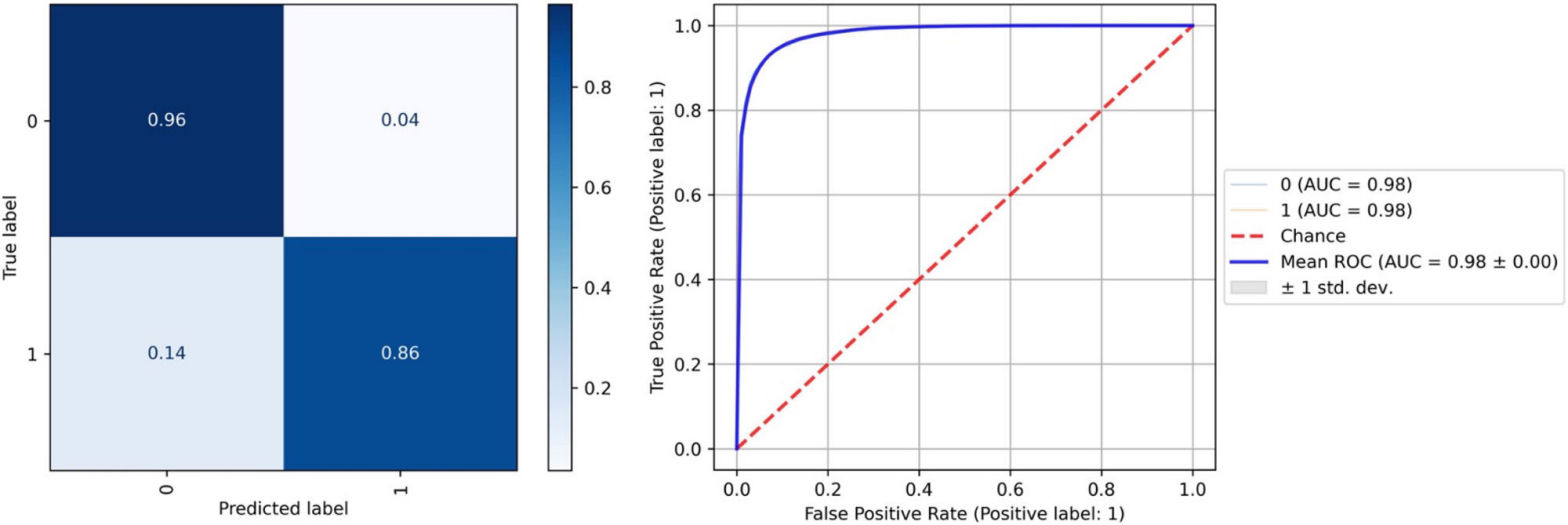
IDH Mutation Classifier Performance. Confusion matrix and ROC for the best performing random forest classifier of IDH mutation (1 = mutant, 0 = wildtype). The classifier could predict IDH mutation with an accuracy of 93%.

Finally, another relevant classification task is the IDH mutation. The best-performing classifier for IDH mutation was a random forest model (150 trees, all available samples per class, 2 pixels per sample, sqrt(n_features) features per tree, Shannon entropy splitting criterion). The average accuracy was 93% in classifying between IDH-mutant and IDH-wildtype tumors. Figure 8 shows this classifier’s confusion matrix and multiclass ROC.

## Discussion

In this paper, four ML-based classifiers were developed for tissue types, IDH mutation, tumor margins, and glioma WHO grades. Each classifier achieved high accuracy. The margin classification accuracy of 85.7% outperforms that of Leclerc et al.^58^ (77% on 50 samples), likely due to the use of much more data. This represents the accuracy of the model of differentiating tumor (solid tumor and infiltration zone) from healthy non-tumorous tissue (reactive brain altered tissue). Our classifier also used fluorophore abundances instead of PCA. Data from 891 hyperspectral measurements of 184 patients was utilized, corresponding to up to 15000 spectra for a given test. Furthermore, the tissue type and WHO grade classifications performed well. The classifiers achieved an average test accuracy of 87.3% when classifying between tissue types and 89.5% when classifying between higher-level groupings of tissue type.

A possible limitation of the accuracies presented in this study is that some of the training data might be noisy if the automatic tumor segmentation did not work well. This is especially relevant for very small, oddly shaped biopsies or ones with a large splash of fluid next to the tumor, which tended to show up very brightly in the fluorescence images. The masks were checked manually, but some background inclusion was inevitable. Current work explores using convolutional neural networks (CNNs) to improve the labeling process. Another source of noise in the labels is described in the Methods and Results sections, as inaccurate or uncertain labeling could decrease training effectiveness. For instance, the delineation between the margin classes is not entirely straightforward, and some tumor types are no longer distinguished since the 2021 WHO classification. This was seen between Figs. 5 and 6. Future work should be carried out to replicate the results in different classes, as defined in the 2021 WHO classification system.

The results could likely be improved with more data on the less frequent tumor types (e.g., APXA, ganglioglioma). For the MLP and random forest models, there was a strong trend of increasing accuracy as we increased the amount of training data. This could be due to the reduced number of labels to classify, resulting in a lower probability of error, or because the training process improved with an increased amount of data. It is likely a combination of both, but it is promising that with continued data collection, we will likely be able to improve classification accuracy.

Furthermore, looking at individual spectra or a few randomly selected ones per biopsy loses spatial information, which might be valuable for the presented classification tasks and the initial calculation of fluorophore abundances. Hence, these tasks could benefit from a convolutional approach that considers spatial information and translational invariance. Future work will explore inputting the hyperspectral data cubes directly into a CNN^62^. In general, we have only considered relatively simple, off-the-shelf models, so accuracy may be improved by considering other learning algorithms. In our tests, random forest models outperformed MLP models for every task. However, with more data or more careful design of the neural network architecture and hyperparameters, a neural network would be expected to outperform the random forests. This is left for future work as this paper focuses on establishing the feasibility of a fluorescence-based approach to tumor classification.

IDH mutations are oncogenic drivers in gliomas and act as a relevant prognostic marker for these tumors^63^. Thus, having this information during surgery could be valuable. In the context of eloquence, a surgeon may adopt a less aggressive approach in younger patients upon identifying an IDH-mutant oligodendroglioma. In the case of an IDH-wildtype tumor, the survival chances would increase relevantly by maximizing resection, and thus an aggressive surgery would be warranted. Our model can differentiate mutations in IDH with an accuracy of 93%. This is promising for future intraoperative applications.

The labeling of biopsies assumes that the entire biopsy belongs to a particular class. This is a reasonable assumption as the biopsies are small (typically 4–10 mm diameter), carefully removed, and thus relatively homogeneous. In general, however, the tissue is very heterogeneous, and the distinction between classes is blurred. This could introduce noise into the margin classification training data. For example, a pixel from a biopsy labeled solid tumor might be in a small region of infiltrating tumor and thus be incorrectly labeled. This could partially explain why the classification accuracy is only 85.7%. The only way to improve this would be to register the fluorescence image spatially with the histopathology to obtain a pixel-for-pixel map of class labels. However, the histopathological assessment is made from thin biopsy slices in unknown orientations and planes, so spatial registration is infeasible with the current setup and constitutes future work.

In addition, heterogeneity is an issue not only for an ML classifier but also for pathologists. The categorization of tumor margins can be subjective, and there is an intra-observer variability among different pathologists and centers, which affects the testing dataset^64,65^. Thus, it is likely that the classification model presented in this paper would perform better if pathologists applied a standardized, quantitative measure to distinguish between different tissue regions. With this in mind, the classification accuracy achieved at 85.7% is very good. More rigorous labeling of margins could be performed in the future by having several pathologists from different centers label the data.

The performance of the classifiers may also be affected by the accuracy of the spectral unmixing, particularly the method and the basis spectra used. This paper used the spectra described previously with non-negative least squares unmixing^35^. However, other spectra have also been proposed for brain tumor fluorescence^66^. The effectiveness of both sets of spectra is evaluated in a recent paper^67^, which also explores the unmixing algorithm, including considerations of sparsity and the underlying probabilistic models. Therefore, future work should extend the analysis presented in this paper to see if new unmixing approaches can improve the accuracy of the classifiers.

Finally, though PCA was used in this paper primarily for visualization, analysis of information loss, and comparison to previous work^58^, it should not be used as an alternative for spectral unmixing. PCA is non-unique, so the factors can be rotated to produce a set of axes that is equivalently optimal from the PCA perspective but which provides an entirely different spectral unmixing result. Instead, independent component analysis (ICA) may be more suitable, assuming the different fluorophores are statistically independent^68^. As a result, the PCA components do not match the shape of the basis spectra, except for the first one, which resembles PpIX_634_ (see Supplementary Fig. 1). Additionally, the fact that the classification results with PCA were worse than those with spectral unmixing could be due to several factors. The scaling of the fluorophore abundances varied greatly, which PCA does not capture well. Furthermore, PCA requires zero-mean data and thus subtracts the mean from the data as one of the first processing steps. However, spectral data is inherently non-negative, leading to unphysical and inaccurate representations.

This paper has explored the effect of different neurosurgically relevant categorizations of brain tumors and tissue on the five previously-characterised^35^ fluorescence emission spectra. At least four of five fluorophore abundances were found to vary statistically significantly (*p* < 0.01) among tumor margins, WHO grades, and tissue types. To test the predictive value of the unmixing, we introduced four different ML-based classifiers for tissue type, tumor margins, WHO grades, and IDH status. Each classifier achieved high accuracy, thus promising practical utility for similar systems in the future and demonstrating the differential expression of the fluorophores in different tissue classes and tumors. Together with the fact that the five mathematically optimal PCA-derived components matched very closely to the physically justified fluorophores, this also shows the value and accuracy of the five fluorophores as biomarkers. Moreover, it shows potential for automatic intraoperative classification systems in fluorescence-guided neurosurgery in the future.

### Supplementary information

Supplementary Information

Description of Additional Supplementary Files

Supplementary Data 1

Supplementary Data 2

Reporting Summary

## Data Availability

Patient data cannot be made publicly available for privacy reasons, but spectral data may be shared with individuals at reasonable request. The raw results from the ML models from which Figs. 4–8 were generated are included in Supplementary Data 1.

## Code availability

The code is not made publicly available because it primarily handles our specific data. The algorithms used (random forests, multi-layer perceptrons, etc.) were implemented in SciKit-Learn and are available open source at scikit-learn.org or any similar library. However, the code may be made available upon reasonable request.
