# Supplementary material for: Towards machine learning-based quantitative hyperspectral image guidance for brain tumor resection
Source: Commun Med (Lond). 2024 Jul 4;4:131. doi: 10.1038/s43856-024-00562-3 (PMC11224305; doi:10.1038/s43856-024-00562-3)
Supplement: Supplementary file 1 — Supplementary Information [file 43856_2024_562_MOESM1_ESM.pdf]

Towards Machine Learning-based Quantitative Hyperspectral Image Guidance for Brain Tumor Resection

Supplementary Information

David Black, Declan Byrne, Anna Walke, Sidong Liu, Antonio Di Ieva, Sadahiro Kaneko, Walter Stummer, Tim Salcudean, Eric Suero Molina

**Fluorophore**	**Pairs with $p > 0.05$**	**p-Values**
Lipofuscin	WHO grade II and III	0.08
Lipofuscin	Meningioma and Medulloblastoma	0.09
Flavin	Oligodendroglioma and Diffuse Astrocytoma	0.24
Flavin	Solid Tumor and Infiltrative Zone	0.06

Supplementary Table 1: **Non-Significant Fluorophore Differences Between Tissue Types**
Two-sample Kolmogorov-Smirnov test for significant differences between tissue types for each fluorophore. All pairs of classes not listed in the table varied significantly ($p < 0.05$) for each fluorophore. PpIX abundance differed between all classes.

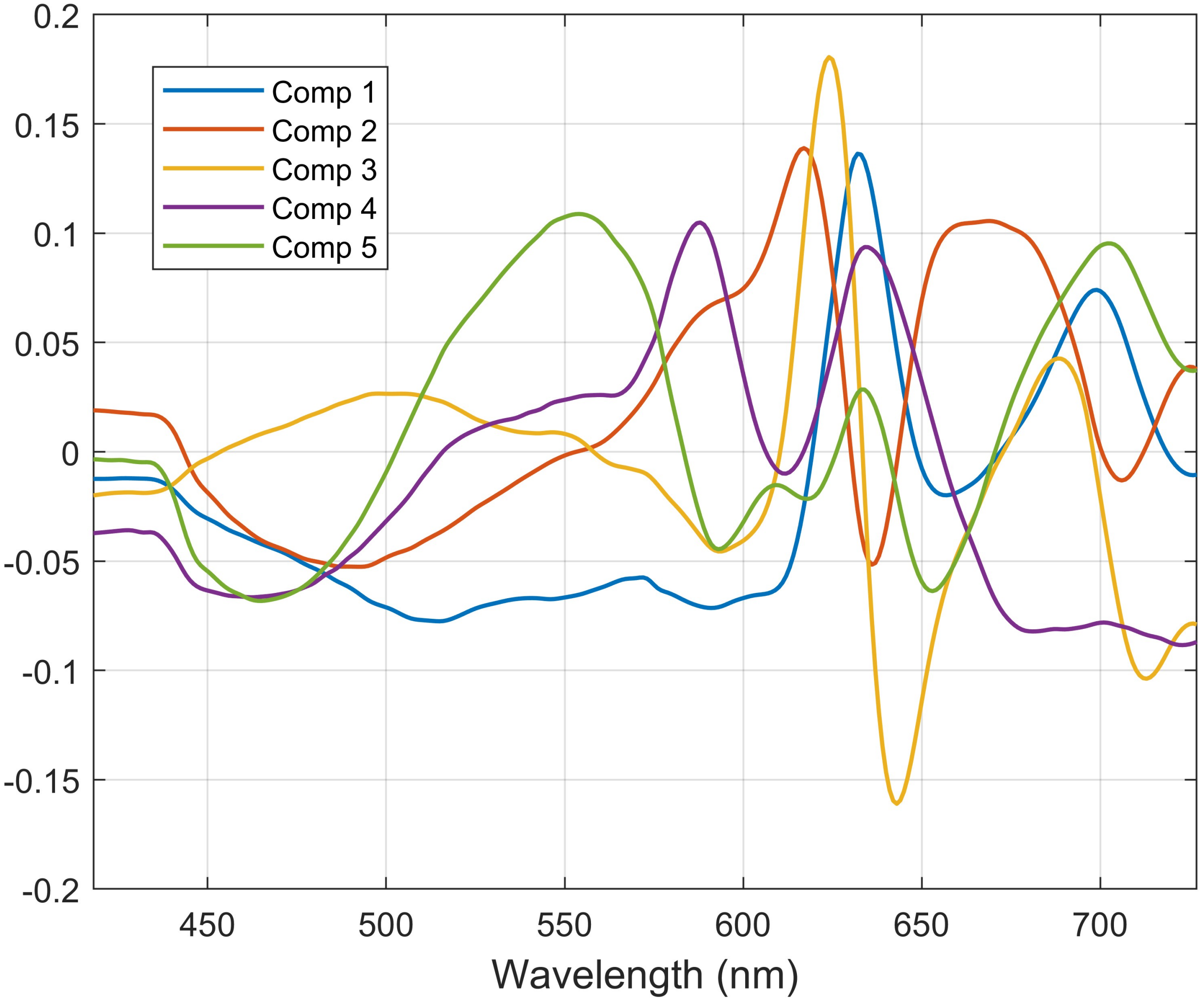

Supplementary Figure 1: **Principal Component Analysis (PCA) of Fluorophore Abundances.**
This figure illustrates the PCA applied to our normalized spectral data. Despite normalization efforts (scaling all spectra to have a magnitude of 1), the analysis reveals significant limitations in the PCA approach for this dataset. Notably, the scaling issue is evident as the representation is disproportionately skewed towards PpIX due to strong measurements from this fluorophore, leading to an underrepresentation of other fluorophores. Additionally, the PCA components exhibit negative values, which contradicts the physical reality of spectral data that is inherently non-negative. These findings underscore the inadequacy of PCA for capturing the varying scales of fluorophore abundances and its unsuitability for handling spectral data, which does not conform to the zero-mean assumption required by PCA. Thus, all classifiers performed better with unmixed fluorophore abundances than with PCA components.

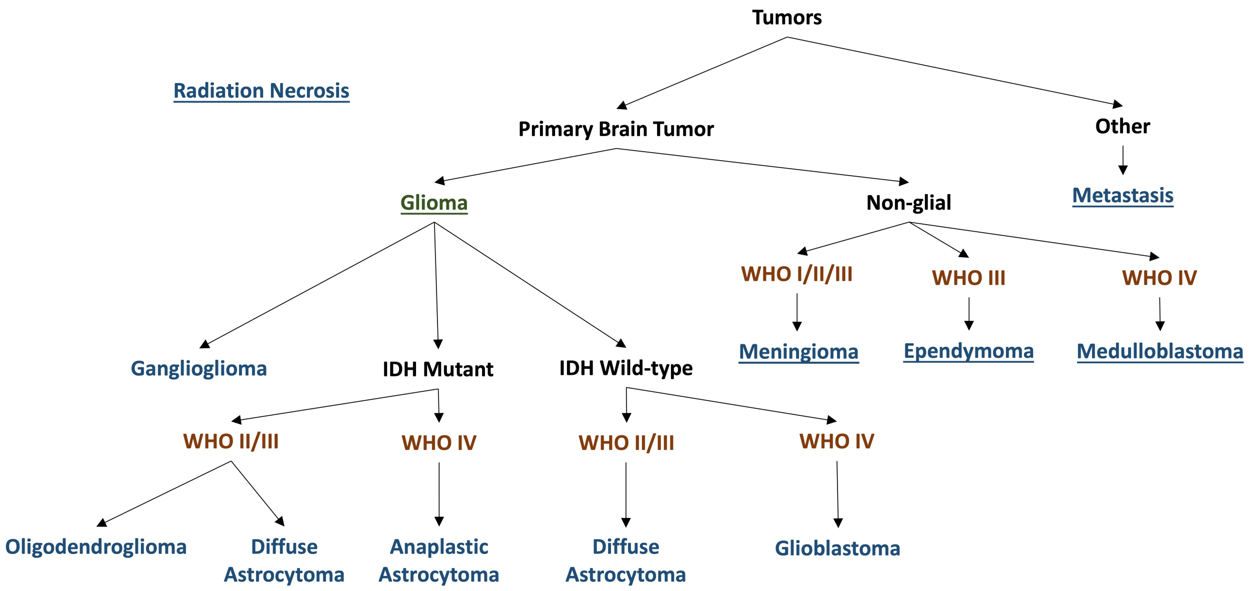

Supplementary Figure 2: **Taxonomy of tissue types considered in this study.**
As many tumors were measured before 2021, this partly uses the 2016 classification. WHO grade (orange) is shown in the WHO Grade Section of the Results. Tissue type classification (blue) and higher-level group classification (underlined) are described in Tissue Type Section of the Results.

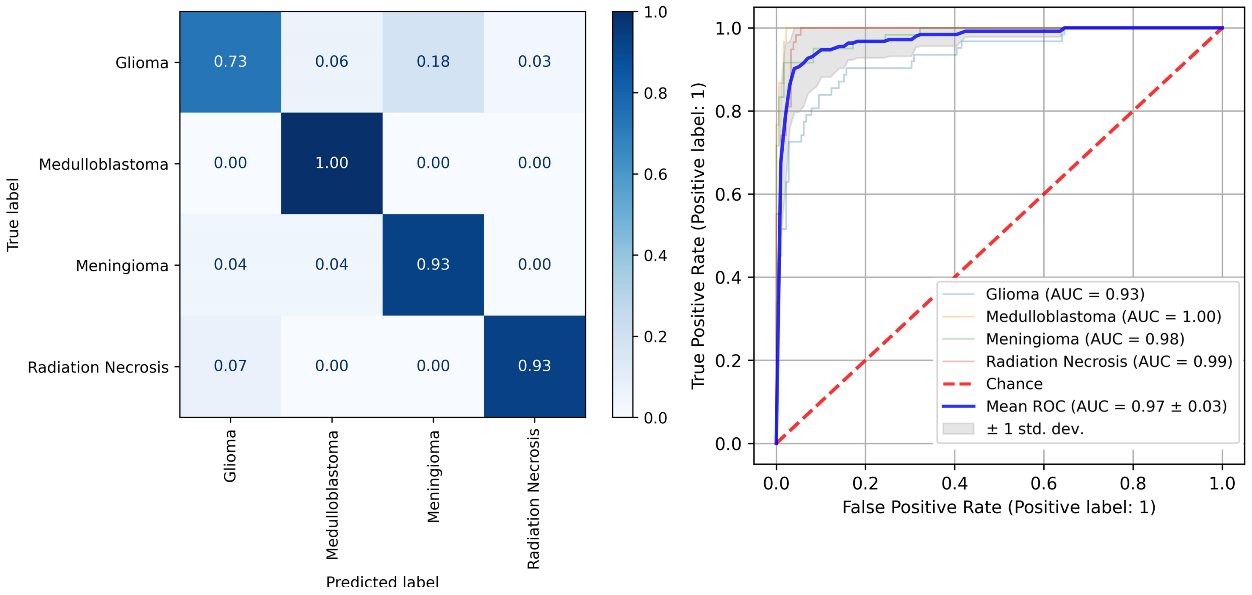

Supplementary Figure 3: **Tissue Type Group Classifier Performance**
Confusion matrix and ROC for best-performing classifier of tissue type groups.
