# Supplementary material for: Towards machine learning-based quantitative hyperspectral image guidance for brain tumor resection
Source: Commun Med (Lond). 2024 Jul 4;4:131. doi: 10.1038/s43856-024-00562-3 (PMC11224305; doi:10.1038/s43856-024-00562-3)
Supplement: Supplementary file 2 — Description of Additional Supplementary Files [file 43856_2024_562_MOESM2_ESM.pdf]

Description of Additional Supplementary Files

File name: Supplementary Data 1

File Description: the raw results.

File name: Supplementary Data 2

File Description: all p values.
