# Supplementary material for: Towards machine learning-based quantitative hyperspectral image guidance for brain tumor resection
Source: Commun Med (Lond). 2024 Jul 4;4:131. doi: 10.1038/s43856-024-00562-3 (PMC11224305; doi:10.1038/s43856-024-00562-3)
Supplement: Supplementary file 5 — Reporting Summary [file 43856_2024_562_MOESM5_ESM.pdf]

nature portfolio

Corresponding author(s): Eric Suero Molina

Last updated by author(s): Apr 18, 2024

Reporting Summary

Nature Portfolio wishes to improve the reproducibility of the work that we publish. This form provides structure for consistency and transparency in reporting. For further information on Nature Portfolio policies, see our Editorial Policies and the Editorial Policy Checklist.

Statistics

For all statistical analyses, confirm that the following items are present in the figure legend, table legend, main text, or Methods section.

n/a	Confirmed	
☐	☒	The exact sample size (*n*) for each experimental group/condition, given as a discrete number and unit of measurement
☐	☒	A statement on whether measurements were taken from distinct samples or whether the same sample was measured repeatedly
☐	☒	The statistical test(s) used AND whether they are one- or two-sided<br>*Only common tests should be described solely by name; describe more complex techniques in the Methods section.*
☐	☒	A description of all covariates tested
☐	☒	A description of any assumptions or corrections, such as tests of normality and adjustment for multiple comparisons
☐	☒	A full description of the statistical parameters including central tendency (e.g. means) or other basic estimates (e.g. regression coefficient) AND variation (e.g. standard deviation) or associated estimates of uncertainty (e.g. confidence intervals)
☐	☒	For null hypothesis testing, the test statistic (e.g. *F*, *t*, *r*) with confidence intervals, effect sizes, degrees of freedom and *P* value noted<br>*Give P values as exact values whenever suitable.*
☒	☐	For Bayesian analysis, information on the choice of priors and Markov chain Monte Carlo settings
☐	☒	For hierarchical and complex designs, identification of the appropriate level for tests and full reporting of outcomes
☐	☒	Estimates of effect sizes (e.g. Cohen's *d*, Pearson's *r*), indicating how they were calculated

Our web collection on statistics for biologists contains articles on many of the points above.

Software and code

Policy information about availability of computer code

Data collection	The data was collected and processed using custom scripts written in National Instruments LabView software and MATLAB respectively.
Data analysis	Data analysis was performed using Python, especially with the libraries SciPy, NumPy, and SciKitLearn

For manuscripts utilizing custom algorithms or software that are central to the research but not yet described in published literature, software must be made available to editors and reviewers. We strongly encourage code deposition in a community repository (e.g. GitHub). See the Nature Portfolio guidelines for submitting code & software for further information.

Data

Policy information about availability of data

All manuscripts must include a data availability statement. This statement should provide the following information, where applicable:

- Accession codes, unique identifiers, or web links for publicly available datasets
- A description of any restrictions on data availability
- For clinical datasets or third party data, please ensure that the statement adheres to our policy

The data was collected from patients and is not available in the public domain, but may be made available to individuals upon a reasonable request.

March 2021

Human research participants

Policy information about studies involving human research participants and Sex and Gender in Research.

Reporting on sex and gender	It is unknown but highly unlikely that patient sex affects the classification of brain tumors by their fluorescence. The differences were not considered in this study, but data from a large and diverse group of patients were used so the results should generalize well.
Population characteristics	The population consisted of a diverse range of 184 adult patients undergoing brain surgery for resection of the following tumor types: Pilocytic astrocytoma (n=5), diffuse astrocytoma (n=57), anaplastic astrocytoma (n=51), glioblastoma (n=410), grade II oligodendroglioma (n=24), ganglioglioma (n=4), medulloblastoma (n=6), anaplastic ependymoma (n=8), anaplastic oligodendroglioma (n=4), meningioma (n=37), metastasis (n=6), radiation necrosis (n=20).
Recruitment	Patients undergoing surgery in our institution for the above-mentioned pathologies, where surgery was indicated in a multidisciplinary team meeting, regardless of this study, were consented to and recruited in this study.
Ethics oversight	Ethical Committee of the University of Münster

Note that full information on the approval of the study protocol must also be provided in the manuscript.

Field-specific reporting

Please select the one below that is the best fit for your research. If you are not sure, read the appropriate sections before making your selection.

☒ Life sciences ☐ Behavioural & social sciences ☐ Ecological, evolutionary & environmental sciences

For a reference copy of the document with all sections, see nature.com/documents/nr-reporting-summary-flat.pdf

Life sciences study design

All studies must disclose on these points even when the disclosure is negative.

Sample size	891 hyperspectral measurements from 184 patients constitute the approximately 550,000 measured spectra that were used in this study. This is by far the largest and most diverse hyperspectral fluorescence dataset in neurosurgery that we know of and is therefore ideal for machine learning approaches.
Data exclusions	8 samples of anaplastic ependymoma were excluded because they are rare but easy to see so fluorescence is not commonly used.
Replication	A test set of approximately 20% of the data was withheld during training and validation and used to test the developed models. These results are presented in the paper.
Randomization	N/A. This paper aims to classify or predict various factors based on hyperspectral measurements. Thus, all available data was used and covariates are welcome as they allow the algorithms to learn better and more robustly.
Blinding	N/A. The analysis was performed using computer algorithms so blinding of the researcher is not relevant.

Reporting for specific materials, systems and methods

We require information from authors about some types of materials, experimental systems and methods used in many studies. Here, indicate whether each material, system or method listed is relevant to your study. If you are not sure if a list item applies to your research, read the appropriate section before selecting a response.

Materials & experimental systems

n/a	Involved in the study	
☒	☐	Antibodies
☒	☐	Eukaryotic cell lines
☒	☐	Palaeontology and archaeology
☒	☐	Animals and other organisms
☐	☒	Clinical data
☒	☐	Dual use research of concern

Methods

n/a	Involved in the study	
☒	☐	ChIP-seq
☒	☐	Flow cytometry
☒	☐	MRI-based neuroimaging

Clinical data

Policy information about clinical studies

All manuscripts should comply with the ICMJE guidelines for publication of clinical research and a completed CONSORT checklist must be included with all submissions.

Clinical trial registration	N/A
Study protocol	N/A
Data collection	N/A
Outcomes	N/A
